# A Systematic Review of the Development and Implementation of Needs-Based Palliative Care Tools in Heart Failure and Chronic Respiratory Disease

**DOI:** 10.3389/fcvm.2022.878428

**Published:** 2022-04-13

**Authors:** Amy Waller, Breanne Hobden, Kristy Fakes, Katherine Clark

**Affiliations:** ^1^Health Behaviour Research Collaborative, College of Health Medicine and Wellbeing, University of Newcastle, Callaghan, NSW, Australia; ^2^Hunter Medical Research Institute, New Lambton Heights, NSW, Australia; ^3^Northern Sydney Local Health District (NSLHD) Supportive and Palliative Care Network, St Leonards, NSW, Australia; ^4^Northern Clinical School, The University of Sydney, Darlington, NSW, Australia; ^5^Northern Sydney Cancer Centre, Royal North Shore Hospital, St Leonards, NSW, Australia

**Keywords:** palliative care, lung disease, heart failure, needs assessment, psychometrics

## Abstract

**Background:**

The impetus to develop and implement tools for non-malignant patient groups is reflected in the increasing number of instruments being developed for heart failure and chronic respiratory diseases. Evidence syntheses of psychometric quality and clinical utility of these tools is required to inform research and clinical practice.

**Aims:**

This systematic review examined palliative care needs tools for people diagnosed with advanced heart failure or chronic respiratory diseases, to determine their: (1) psychometric quality; and (2) acceptability, feasibility and clinical utility when implemented in clinical practice.

**Methods:**

Systematic searches of MEDLINE, CINAHL, Embase, Cochrane and PsycINFO from database inception until June 2021 were undertaken. Additionally, the reference lists of included studies were searched for relevant articles. Psychometric properties of identified measures were evaluated against pre-determined and standard criteria.

**Results:**

Eighteen tools met inclusion criteria: 11 were developed to assess unmet patient palliative care needs. Of those, 6 were generic, 4 were developed for heart failure and 1 was developed for interstitial lung disease. Seven tools identified those who may benefit from palliative care and include general and disease-specific indicators. The psychometric qualities of the tools varied. None met all of the accepted criteria for psychometric rigor in heart failure or respiratory disease populations. There is limited implementation of needs assessment tools in practice.

**Conclusion:**

Several tools were identified, however further validation studies in heart failure and respiratory disease populations are required. Rigorous evaluation to determine the impact of adopting a systematic needs-based approach for heart failure and lung disease on the physical and psychosocial outcomes of patients and carers, as well as the economic costs and benefits to the healthcare system, is required.

## Introduction

Practice guidelines from multiple societies and international policy documents emphasize the importance of delivering equitable and appropriate palliative care to people diagnosed with advanced heart failure (HF) and chronic respiratory diseases, such as chronic obstructive pulmonary disease (COPD) and interstitial lung disease (ILD) [e.g., (1–5)]. People living with these progressive conditions will eventually experience physical function decline, as well as changes to their psychological, social and spiritual functioning and wellbeing (6–8). Despite comparable mortality rates and symptom burden, fewer people with these conditions are referred to palliative care services and when they are it is typically later compared to those with a cancer diagnosis (9–11). For instance, a Canadian retrospective population-based study reported that significantly fewer patients with COPD received specialist palliative care (SPC) compared to those with lung cancer (20 vs. 57%) (12). A UK population based study of over 92,000 patients with COPD found only 7.8% of the cohort received SPC (13). A systematic review of studies with patients with ILD reported palliative care involvement ranging from 0 to 38% (14). Similar data have been reported for patients with HF in the USA (15, 16), UK (17), Australia (18), Canada (19) and Europe.

A range of patient-, provider- and system- related factors contribute to non-referrals, late or crisis referrals to palliative care for patients with chronic HF and chronic respiratory disease. Patients and families have identified denial, misperception about the potential benefits and purpose of palliative care, and negative previous experiences with services (20, 21). While some providers report feeling comfortable providing a palliative approach (22), for others there is uncertainty about the role of palliative care and when this approach should be introduced (23, 24). Health care providers' poor recognition of their patient's palliative care needs can be impacted by time constraints, a lack of education or training, and awareness or availability of standardized tools and referral pathways (20, 22–25). Some health care providers perceive palliative care is not as useful for non-malignant conditions or that SPC services prioritize patients with cancer (26). Limited availability of SPC services and workforce shortages also limit timely referrals (20, 23, 25, 27). Poor integration of palliative care and cardiology and respiratory services has been reported (28).

In addition to the aforementioned factors, one of the most pertinent barriers to palliative care referrals remains the ongoing reliance on diagnosis and estimated prognosis as the main trigger for palliative care referral (9, 27). Diagnosis-based approaches have contributed to the over-representation of cancer patients in SPC services. Prognosis as a prompt for palliative care is also problematic, given the unpredictable trajectory (29) and evidence of inaccurate estimates by clinicians for patients with progressive chronic diseases (9). For instance, respiratory providers and general practitioners report reliance on the “surprise question” (SQ), which asks clinicians “Would you be surprised if this patient died in the next 12 months?”, to promote referrals (30), despite reports of poor to modest prognostic accuracy across studies of patients diagnosed with organ failure, cancer and those attending general practice (31, 32).

A shift from prognosis and diagnosis-based approaches to a needs-based approach for guiding delivery of care has been advocated by international bodies such as Palliative Care Australia and the World Health Organization (WHO). Underpinning this approach is the timely recognition of needs and the delivery of holistic care by non-palliative care specialists to all those with a life-limiting illness. Studies highlight high levels of unmet needs across physical, psychological, social, practical and information domains for patients with HF and COPD, and their carers (33, 34). Therefore, a key component to support the successful integration of a needs-based approach in clinical practice requires the rigorous development, testing and implementation of tools that can accurately assess palliative care needs across a range of settings and diseases (35). Needs assessment tools have been broadly categorized into two groups: those developed to assist in the early identification of individuals who would benefit from palliative care; and those developed to identify and monitor unmet palliative and supportive care needs (35). Factors to consider in tool selection include the: (i) purpose, context and target population being assessed; (ii) the acceptability of the tool to patients, families and health care professionals; (iii) and the psychometric qualities of the instrument (35). Introduction of these tools requires a structured approach, given the potential impact on patients and services, with a particular emphasis on acceptability, feasibility and cost-effectiveness.

## Aims

This systematic review examined palliative care needs tools for people diagnosed with advanced HF or chronic respiratory diseases, to determine their: (1) psychometric quality; and (2) acceptability, feasibility and clinical utility when implemented in clinical practice.

## Methods

### Literature Search

The electronic databases Medline, CINAHL, Embase, Psycinfo and Cochrane were searched using a combination of Medical Subject Headings (MeSH) and keywords (see Supplementary Table 1 for the full search strategy). Major search terms included: “needs assessment,” “unmet needs,” “palliative care,” “hospice and palliative care,” in addition to general and more specific search terms for advanced HF and the major types of chronic respiratory disease. Searches were limited to studies published from the earliest records for each database until June 2021 and studies conducted with humans. The reference lists of included studies and the reference lists of relevant review articles were also manually searched to identify any additional studies.

### Inclusion and Exclusion Criteria

Studies were included if they: (i) focused on people diagnosed with HF or chronic respiratory disease (e.g., COPD, ILD); (ii) included a tool that aimed to identify individuals for whom a palliative approach is required or assess palliative care needs; (iii) examined psychometric properties, acceptability, feasibility or clinical utility of a palliative care tool; and (iv) included primary collected data. Studies that included a heterogeneous sample of patients including HF and/or chronic respiratory disease patients, were included if they reported outcomes separately for the target population(s); or reported on a sample comprising at least 50% of the target populations.

Studies were excluded if they: (i) were published in a language other than English; (ii) examined tools assessing aspects of health or care other than needs, such as symptoms (e.g., Edmonton Symptom Assessment Scale, St George Respiratory Questionnaire), quality of life (e.g., Minnesota Living with Heart Failure), functional status (e.g., Australian Karnofsky Performance Scale), satisfaction with care (e.g., Quality Care Questionnaire- Palliative Care); (iii) focused on one needs domain, and (iv) were reviews, case studies, commentaries, theses, conference abstracts, protocols or editorials.

### Study Screening

**Figure 1:**
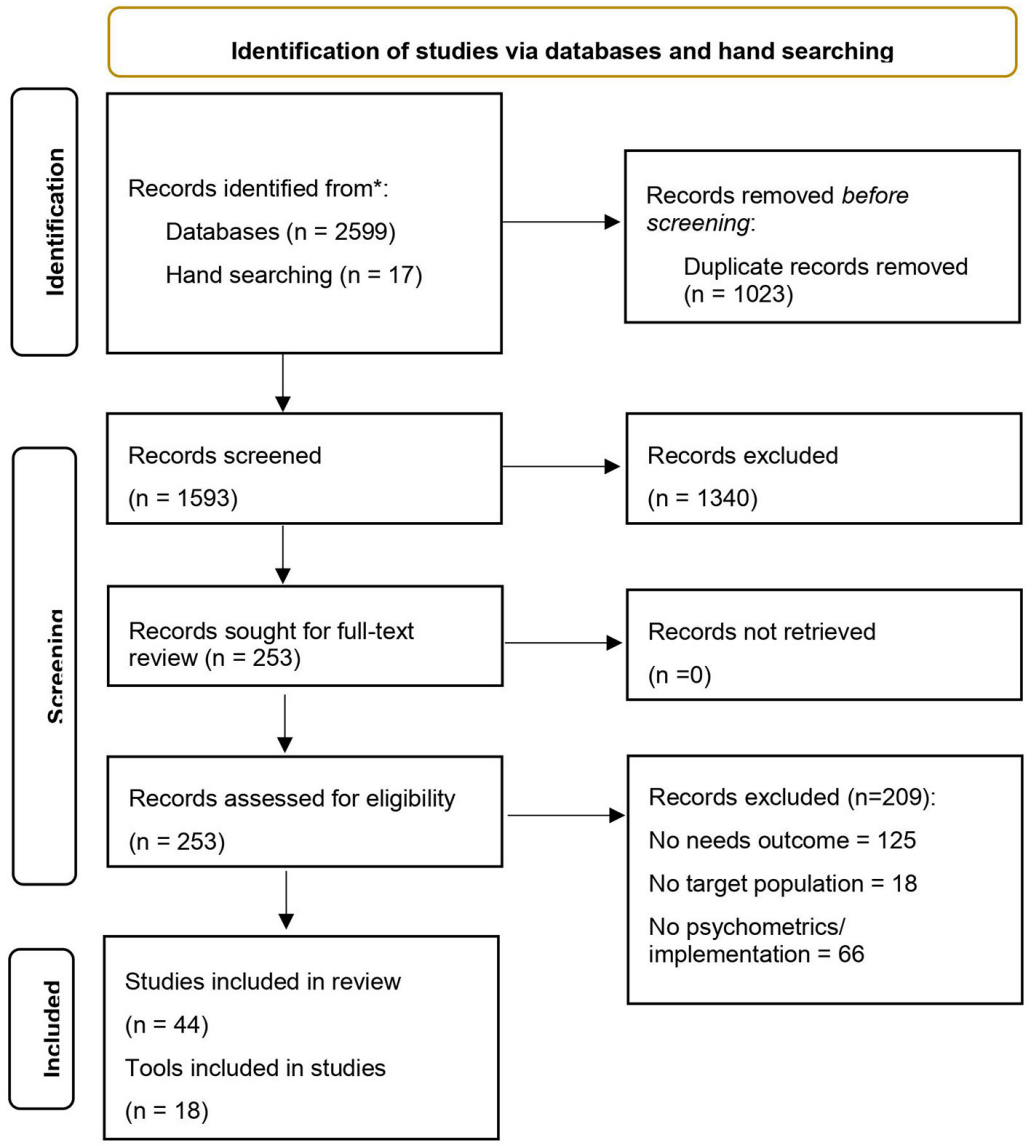
PRISMA flow diagram.

Article screening and coding was conducted using the reference management system Covidence. Following removal of duplicate citations, reviewers (AW, BH, KF) independently screened the titles and abstracts of all retrieved studies according to inclusion and exclusion criteria. Discrepancies were resolved by consensus between reviewers, or where there was insufficient detail available to exclude on the basis of study title and abstract, these studies progressed to full-text review. Pairs of reviewers (AW, BH and KF) independently assessed full-text articles for their eligibility for inclusion. Reasons for excluding studies at full-text review were documented (Figure 1 PRISMA flow diagram of included studies). If discrepancies between reviewers for study inclusion could not be resolved by consensus, a field expert (KC) was consulted as a fourth reviewer to determine inclusion. Three authors (AW, BH and KF) undertook data extraction. Discrepancies were resolved by consensus.

### Data Extraction

#### Characteristics for Studies Examining Psychometric Properties of Existing Tools

Study characteristics and the sample used to develop and/or validate each of the included tools were extracted for all psychometric articles: (a) population; (b) country and setting, (c) purpose; (d) tool completion; (e) domains and items; (f) question format and (g) psychometrics. The psychometric properties were evaluated against pre-determined and generally accepted criteria including: reliability (internal consistency, inter-rater reliability and test-retest); validity (face, content, construct, and criterion); responsiveness; and cross-cultural adaptation, summarized in Table 2.

#### Characteristics of Studies Examining Implementation of Existing Tools

Study data extracted from each study implementing the included tools: (a) study design and aims; (b) setting and sample characteristics; (c) evaluation/intervention strategies; and (d) summary of outcomes.

### Data Synthesis

A narrative approach was taken to synthesis the psychometric and implementation data of studies examining the included tools.

## Results

### Search Results

An overview of the search results and study coding process is outlined in Figure 1 using the Preferred Reporting Items for Systematic Reviews and Meta-Analysis (PRISMA) flow diagram. The initial search yielded 2,616 articles. After removing 1,023 duplicates, 1,593 articles were included in the title and abstract screen. A total of 253 studies were included in the full-text review, of which, 44 met inclusion criteria (30 studies of 18 tools assessing psychometric properties with the target populations; 14 implementation studies) (see Tables 1, 2).

**Table 1:** Study and sample characteristics used to develop and/or psychometrically test identified tools.

**Measure and author(s)**	**Population**	**Country, setting**	**Completion**	**Domains and items**	**Question format**	**Psychometrics**
**Identify those in need of palliative approach**						
**CriSTAL** Criteria for screening and triaging to appropriate alternative care (36)	Older; any condition; high probability dying in ≤ 3 months.	Australia USA Netherlands Denmark Ireland Acute	Provider	**29 indicators**: Age≥65; ED admission; ≥2 deterioration criteria, frailty with ≥2 criteria; early warning score>4; presence ≥1 comorbidities; NH placement; cognitive impairment; repeat hospitalization/ICU; abnormal ECG; proteinuria	Presence/absence: “Yes,” “No”	Face, content validity (36) Predictive validity (mortality, palliative care referral): retrospective (37, 38); prospective (39) (*additional articles in press*)
**GSF PIG** Gold Standard Framework Prognostic Indicator Guide (40)	Heart disease COPD	UK Tertiary care	Providers (to determine palliative care needs)	**8 items:** The Surprise Question (SQ); General indicators of decline; Specific clinical indicators related to certain conditions.	Presence/absence: “Yes,” “No,” “Don't know”	Predictive validity (41, 42) Sensitivity and specificity for COPD; sensitivity and specificity for COPD and HF (mixed patient sample) (43)
**NECPAL** Palliative Needs World Health Organization Collaborating Center (English translation) (40, 42)	Heart Failure (NECPAL-HF) Respiratory conditions	Spain Tertiary care	Provider	**17 indicators**: SQ, Requests for PC; General indicators (Functional decline, weight decline; Geriatric syndromes; Psychological adjustment Comorbidities Resources/ admissions) Disease-specific indicators	Presence/absence: “Yes,” “No”	Predictive validity (HF) (44) Content validity
**P-Cares** Palliative care and rapid emergency screening Tool (45)	Any ED patient with life limiting illness	USA Emergency Department	Provider Time taken: 1.8 min (average)	Presence life limiting illness: advanced COPD, advanced HF advanced dementia, cancer, end stage renal, end stage liver, septic shock, chance of accelerated death PC needs: frequent visits, uncontrolled symptoms, functional decline, uncertain GOC/care distress, SQ	Presence/absence: “Yes,” “No” Score 1+ life-limiting illness and 2+ PC needs indicates PC referral	Inter-rater reliability (46) Face, content validity (45) Criterion validity Acceptability (46) Predictive validity, prognostic utility (47)
**ProPal-COPD** (48)	COPD	Netherlands Hospital	Patients Providers	**2 patient reported indicators**: Medical Research Council dyspnea (MRC dyspnea); Clinical COPD Questionnaire (CCQ) 10 questions and 3 domains: symptoms, functional status and mental state. **Provider indicators**: SQ, 5 markers COPD severity, presence of comorbidities.	MRC dyspnea: 1 to 5. Higher scores = more severe dyspnea. CCQ: Total score = 6. Higher score = worse health status. Total score > – 1.362 = a high probability for death within 1 year.	Predictive validity (48)
**RADPAC** RADbound indicators for palliative care needs (49)	COPD Heart failure	Netherlands Primary care	Provider (to identify who requires a palliative care assessment)	General indicators (Functional decline, weight decline; patient-reported concerns Hospital admissions) Disease-specific indicators	Presence/absence: “Yes,” “No”	Content validity (49)
**SPICT** Supportive and Palliative Care Indicators Tool (50)	Heart disease	UK Belgium Primary care Hospital	Provider (to identify who requires a palliative care assessment) Time taken: 4–5 min	**6 general, 21 specific indicators** SQ; General (Functional decline, weight decline; hospital admissions); Requests for PC Living in NH; Persistent symptoms; Disease-specific indicators	“Yes,” “No”	Sensitivity and specificity cardiology patients (51) Additional psychometrics available (50, 52–54)
**Assess unmet palliative care needs**						
**CareQol CHF** Care-Related Quality of Life survey for Chronic Heart Failure (55, 56)	Heart failure	Netherlands Any	Patient	**20 items, 3 scales**: social and emotional problems; physical limitations; being in safe hands	In last 2 weeks: “never,” “seldom,” “sometimes,” “often,” “always,” “not applicable.”	Internal consistency (55) Face validity (56) Construct validity (55) Criterion validity (55)
**HFNAQ** Heart Failure Needs Assessment Questionnaire (57)	Heart Failure	Australia Any	Patient Time taken: 10 min	**30 items, 4 domains:** Physical (10 items), Psychological (9 items), Social (8 items), Existential (3 items)	Need for help in last month: 1 (“hardly ever”) to 5 (“always”)	Internal consistency (57) Content validity (57) Construct validity (57) Concurrent validity (57) Discriminant validity (57)
**I-HARP** Identification of patients with HeARt failure with PC needs (58)	Heart Failure	Netherlands Primary Secondary Nursing homes	Provider, (in consultation patient/family) Time taken: 34 min (10–60 mins)	**13 items:** physical, daily activities, information, coping, psychological, culture/religion, social support, finances, future expectations/worries, carer needs. Open ended question: carer need for information	Presence/absence: “Yes,” “No”	Face, content validity (58) Validity and reliability testing planned
**NAT: PD-HF** Needs Assessment Progressive Disease – Heart Failure (59)	Heart Failure [adapted from original NAT: PD-C (60)]	Australia Netherlands Germany Any	Provider Time taken: 5–10 mins (59) Time taken: 26 min (61)	**18 items, 3 domains:** Patient wellbeing Ability of caregiver/family to care for patients Caregiver/Family wellbeing	Level of concern: “none,” “some/potential”, “significant” Provider action to manage concern: “directly managed,” “managed team,” “referral.”	Internal consistency (61) Face, content validity (59, 62) Inter-rater reliability (59, 61, 62) Test-retest reliability (61) Concurrent validity (59) Cultural adaptation (61, 62)
**NAT:PD-ILD** Needs Assessment Progressive Disease for people with Interstitial Lung Disease (63)	ILD [adapted from NAT: PD-C (60)]	UK Any	Provider with patient/carer Time taken: 5–10 min	**22 items, 4 domains:** Red flag symptoms and/or Priority referrals (7) Patient wellbeing (7) Ability of carer to care for patients (6) Carer wellbeing (2) Referral section	Level of concern: “none,” “some/potential,” “significant” Provider action to manage concern: “directly managed,” “managed team,” “referral.”	Test-retest reliability (64) Face, content validity (63, 65) Construct validity (64) Inter-rater reliability (64)
**NEST** Needs near the end-of-life scale (66, 67)	Original: Mixed older: heart failure, renal, stroke, dementia, liver, pulmonary diseases (68) Modified: lung transplant (69)	**USA** Emergency department Outpatient Inpatient	Patient Provider	**Original 13 items:** Financial, Access to care, Social connection, Caregiving, Distress, Spirituality, Sense of purpose, Patient-clinician relationship, Clinician communication, Personal acceptance **Modified version 46 items*****:*** additional 3 cultural items, 1 open-ended and 9 ESAS items.	Care needs at end of life: 0 (“no need”) to 10 (“highest need”); higher scores = higher needs	**Original version:** Feasibility (68) Additional psychometrics available for cancer patients (66, 67, 70) **Modified version** (69) Internal consistency Content validity
**PNAP** Patient needs Assessment in Palliative Care (71)	End stage chronic diseases	Czech Republic Hospital	Patient Time taken: 45 min (average)	**40 items; 7 domains:** Physical symptoms (12) Social area (6) Respect/support from health professionals (5) Meaning of life (6) Autonomy (7) Share emotions (2) Religious needs (2)	Importance: 1 (“not at all important”) to 5 (“very important”) Satisfied: 1 (“not met”) to 5 (“met in full”) Higher score = greater importance/satisfaction	Internal consistency (71) Test-retest reliability Face, content validity Construct validity Convergent validity
**IPOS** Integrated Palliative Outcome Scale (72, 73)	COPD Heart failure (also: Cancer Dementia HIV/AIDS Kidney, Parkinson, Motor Neuron Disease, Multiple Sclerosis)	**UK** Any	Patient, Carer/proxy, Provider versions Time taken: 10 min	**POS 10 items:** Pain and other symptoms, patient anxiety, family anxiety, information, level support, life worth, self-worth, waste time, personal affairs. Patients open ended item to identify main problem; Staff asked performance status **IPOS 17 items, 3 domains**: Physical, Emotional, Communication/Practical Domains; Patients open ended item to identify main problem	**POS**: Problems, quality of life ≤ 3 days; Scales: 0 (“no problem”) to 4 (“overwhelming problem”); higher score = more problems **IPOS:** Problems last 3 days: 0 (“no at all”) to 4 (“overwhelming”). Total Score range 0 to 68; higher values = worse outcome	[see Buasewein et al. and Collins et al. for detailed overviews POS psychometrics (74, 75)] **IPOS (Mixed sample, 7% COPD and HF)** Internal consistency (72) Test-retest reliability (72) Inter-rater reliability (72) Construct validity (72) Face, content validity (76) Responsiveness (72) Further validation in progress
**SCNS-SF34** Supportive Care Needs Survey Short Form (original cancer version) (77)	Cardiovascular disease (78) Cystic fibrosis (79)	Australia Germany USA Any	Patient	**34 items, five domains:** Psychological, health system & information, physical & daily living, patient care & support, and sexuality	Level of unmet need last month: 1 (“no need”), 2 (“satisfied need”), 3 (“low need”), 4 (“moderate need”), 5 (“high need”); higher scores = higher levels of unmet need	Internal consistency (78, 79) Additional psychometrics available - cancer only (77)
**SPARC** Sheffield Profile for Assessment and Referral to Care (80, 81)	Idiopathic pulmonary fibrosis (82) Cancer (81, 83) Stroke (84)	UK Any	Patient Any setting	**45 items:** Communication/ information, Physical, Psychological, Religious and spiritual, Independence and activity, Family and social, Treatment **IPARC version:** 11 items (79)	Level of need last month: 0 (“not at all”) to 3 (“very much”) Desire for help last month: “Yes,” “No” Any score of 3 – referral for further assessment	Internal consistency Face, content validity (81) Convergent and divergent validity Predictive validity (disease progression, mortality) (82)
**Unnamed measure** (85)	Heart Failure COPD Also oncological disease with metastasis	Bulgaria General practice	Carers	Not reported	“Yes,” “No”; multiple choice; Two short answer	Test-retest reliability (85)

### Properties of Identified Tools

#### Purpose, Population and Context

As seen in Table 1, 11 tools have been developed with the primary aim of assessing and monitoring unmet needs across the spectrum of palliative care domains. Generic measures suitable for assessment of needs across a range of chronic diseases included the Integrated Palliative Outcome Scale (IPOS), which is one of the most established and well-validated tools in palliative care, as well as the Needs near the end-of-life scale (NEST); Patient Needs Assessment in Palliative Care (PNAP); Supportive Care Needs Survey Short Form (SCNS-SF34); Sheffield Profile for Assessment and Referral to Care (SPARC) and an unnamed proxy-completed measure. The remaining tools were developed and tested among people diagnosed with HF (Care-Related Quality of Life survey for Chronic Heart Failure [CareQol CHF]; Needs Assessment Tool: Progressive Disease – Heart Failure [NAT: PD-HF]; Heart Failure Needs Assessment Questionnaire [HFNAQ]; and Identification of patients with HeARt failure with PC needs [I-HARP]) or chronic respiratory diseases (Needs Assessment Progressive Disease for people with Interstitial Lung Disease [NAT: PD-ILD]). Two of these tools, the NAT: PD-HF and NAT: PD-ILD, included items that assessed the needs of both patients diagnosed with HF or ILDs and their carers within the same instrument.

The remaining seven tools incorporate broader assessments that include general and disease-specific indicators with the primary aim of identifying people with progressive chronic diseases who are at risk of deteriorating and may benefit from palliative care across a range of settings. These include the Supportive and Palliative Care Indicators Tool (SPICT) for application across care settings (50); the Gold Standard Framework Prognostic Indicator Guide (GSF PIG) tested in tertiary care (40); the RADbound indicators for Palliative Care Needs (RADPAC) tool developed to support general practitioners (GPs) (49); and the Palliative Needs WHO Collaborating Center (NECPAL- CCOMs) tool, adapted from the SPICT and GSF PIG (40). Hospital-specific tools include the Criteria for Screening and Triaging to Appropriate alternative care (CrisTAL) tool for older person likely to die within the next 3 months (36); and the Palliative Care and Rapid Emergency Screening (P- CaRES) tool (45). The ProPal-COPD was developed for application for patients with COPD (48).

#### Reliability

##### Internal Consistency

Eight tools assessed the internal consistency of the scale. Of these, four reported adequate Cronbach's alphas [exceeding 0.70 (96)] for the total scale and each domain (CareQol CHF, HFNAQ, SCNS-SF34 [in cardiovascular population], PNAP). For the IPOS, SCNS-SF34 (in cystic fibrosis population) and NEST, internal consistency was partially confirmed (Cronbach's alpha of <0.70 for at least one domain).

##### Test-Retest Reliability

Only four tools examined test-retest reliability. One met the criteria (k > 0.60) for the total scale and each domain (PNAP); for the remainder (IPOS, NAT: PD-ILD, unnamed measure) test-retest reliability was partially confirmed (k <0.60 for at least one domain).

##### Inter-rater Reliability

Inter-rater reliability was assessed for three tools, including the P-Cares, NAT: PD-HF and NAT: PD-ILD using hypothetical case vignettes and video simulated consultations. Inter-rater reliability was confirmed (IRR cutoff of Gwet's AC1 = 0.8) for the P-Cares. At least moderate agreement was found across all items in the NAT: PD-HF (prevalence and bias-adjusted kappa range 0.54-0.90); while inter-rater reliability was partially confirmed for the NAT: PD-ILD (5/16 items had moderate agreement, 11/16 had fair agreement). Inter-rater reliability was explored for the IPOS using patient-staff and staff-staff ratings of 376 patients receiving palliative care in a range of settings in two countries (72). Kappa scores (at least ≥= 0.4) were reported for 11 of 17 IPOS items.

#### Validity

##### Face and Content Validity

Face and/or content validity was reported for 12 tools. To establish face and content validity, approaches included reviewing previous literature on palliative care needs (CrisTAL, P-Cares, PNAP, RADPAC), adapting items derived from existing tools (CrisTAL, NECPAL, NAT:PD-HF, NAT: PD-ILD, NEST, PNAP, IPOS); and using expert panels and/or focus groups and interviews with health care providers, patients and/or caregivers to derive or refine selected items (CareQol CHF, CrisTAL, NEST, NAT:PD-HF, NAT: PD-ILD, P-Cares, PNAP, IPOS, RADPAC). Some studies employed multiple strategies to select and refine items (HFNAQ, I-HARP, P-Cares, SPARC).

##### Construct Validity

Adequate construct validity was demonstrated for four tools, with mixed results reported for the NAT: PD-HF. Convergent and divergent validity were examined against other existing tools (CareQol CHF, NAT: PD-HF, NAT: PD-ILD, IPOS, PNAP). Factor analysis was performed to examine construct validity (CareQol CHF, IPOS). Construct validity has also been established for original versions of some tools (e.g., POS, NEST, SPARC, SCNS-SF34). While evidence for construct validity in HF and chronic respiratory disease populations were not available for all tools and all disease-specific subscales reviewed, some authors reported that additional data is forthcoming (e.g., IPOS, I-HARP).

##### Criterion Validity

Some tools assessing level of unmet need examined criterion validity through comparison with established measures. Adequate criterion validity was established for the CareQol CHF and P-Cares. Three studies of the NAT: PD-HF demonstrated mixed results in relation to construct and criterion validity (17, 59, 61). Other studies focused primarily on examining the predictive validity of tools used to identify those in need of palliative care, particularly in relation to predicting disease progression, mortality and/or palliative care referral (CrisTAL, GSF-PIG, NECPAL, P-Cares, ProPal-COPD, SPARC and SPICT tools).

#### Responsiveness

There was limited evidence found for tool responsiveness (or sensitivity) to change over time, with only one study examining this psychometric property. A change of 5 points in the total IPOS score was reported to represent a moderate effect size in a mixed palliative population (72).

#### Administration Mode and Acceptability

Nine tools were completed by health care providers, two included both patient and provider assessment, and seven were self-completed by patients and/or their family or carer proxies. Acceptability was typically evaluated by assessing the length of time taken to complete the tool, reading ease and number of missing items. Where reported, average completion time ranged between 2 min (e.g., P-Cares) and 45 min (e.g., PNAP). Readability was reported for the IPOS, however, no further details were provided in relation to how this was examined (90). Only one study reported the proportion of missing items. A non-response rate of 6% was reported for the IPOS questionnaire, a value greater than the 5% threshold for acceptability (90). Respondent feedback was also obtained about ease of use, clarity, and comprehensiveness of the items for some tools. Further evidence of acceptability, feasibility and clinical utility of tools when implemented in clinical practice is summarized below and in Table 2.

**Table 2:** Summary of studies summarizing the implementation of tools to identify and assess palliative care needs.

**Intervention Studies**					
**Tool**	**References, country**	**Study design, aim**	**Setting, Sample**	**Evaluation/Intervention**	**Summary of outcomes**
**Clinical utility (prevalence)**					
GSF-PIG	(86), Australia	**Design:** Prospective cohort study **Aim:** To test the prevalence, recognition and outcomes of patients with PC needs in acute care	**Setting:** University hospital **Sample:** Total *N* = 636 (COPD and HF included)	Criteria for initiation of treatment limitation were created using the clinical criteria from the UK GSF prognostic indicator criteria. Audit of hospital electronic database and patient records in two 24-h periods.	27% (*N* = 171) met GSF criteria, of which 12% had COPD and 6% had HF Age, hospital length of stay, GSF COPD criteria increased likelihood in-hospital treatment limitation Hospital mortality (9.9%), highest in patients with GSF HF criteria (30%)
IPOS (German version)	(87), Germany	**Design:** Cross-sectional, implementation study **Aim:** To test the utility of IPOS in assessing palliative care needs in patients with HF	**Setting:** University hospital **Sample:** *N* = 100 HF inpatients	IPOS completed by patients during hospital admission. Two items assessed the comprehensibility and suitability of IPOS.	Patients reported IPOS was: easy to understand (95%); suitable to assess palliative care needs (91%) 56% patients were suitable for SPC co-management (defined by: 2+ items “overwhelming”, 3+ items “severe”) No significant difference in IPOS total score between NYHA functional class II/III vs. IV, therefore all patients should receive needs assessment
NAT: PD-HF	(17), Australia	**Design:** Prospective cohort study **Aim:** Identify which patients with HF should receive SPC through implementing newly developed PC definition	**Setting: One** community hospital **Sample:** *N* = 272 HF patients (963 assessments)	Index admission assessments including: NAT: PD-HF Prognostic assessments (laboratory, echocardiographic) Physician assessments (physical, AKPS) Medical and drug history Patient measures (QoL, symptom burden, mood disturbance) Carer burden Repeated 4 monthly for 12–21 months	74 (27%) of HF patients had SPC needs Those with SPC need had: worse New York Heart Association class distribution prior to admission; higher % hospitalized in <6months for worsening HF; lower performance status (AKPS); and significant needs on NAT: PD-HF. 24% of those who needed SPC received it
NECPAL-HF	(44), Spain	**Design:** Prospective cohort study **Aim:** To identify HF patients for whom PC may be needed using NECPAL-HF indicators	**Setting:** Ambulatory clinics in three university hospitals **Sample:** *N* = 922 HF patients	NECPAL completed by nurse/physician at a scheduled clinic visit over 4 month period	32.1% (*N* = 297) patients were in need of PC 1 year mortality significantly higher in NECPAL-HF + patients (21.9 vs. 3.8%) The area under the receiver operating characteristics curve for predicting all-cause 1-year mortality was 0.73
NECPAL CCOMS-ICO	(88), Brazil	**Design:** Prospective cohort study **Aim:** To identify the need for palliative care in hospitalized patients with advanced CHF	**Setting:** Hospital **Sample:** *N* = 82 HF patients	NECPAL-HF questionnaire completed by nurse and/or a physician at a scheduled clinic visit over 4 month period	55% in need of PC using NECPAL
SPICT	(51), Belgium	**Design:** Prospective, implementation study **Aim:** To implement and validate SPICT in identifying older hospitalized in need of PC.	**Setting:** Hospital **Sample:** *N* = 209 geriatric patients; *N*=249 cardiology patients	SPICT completed by clinician during hospital admission; Carer contacted 1 year later for survival status and timing death	40% of older people on CUs were SPICT identified. CU SPICT identified patients reported more functional needs and symptoms than SPICT non-identified CU patients. Moderate sensitivity and specificity for CI (0.69 and 0.67 respectively)
**Acceptability, feasibility and effectiveness of implementation**					
IPOS	(89), UK	**Design:** Single-blind RCT **Aim:** To test the impact of the SIPS intervention on clinical and economic outcomes for older people living with chronic non-cancer conditions	**Setting: Four** general practices **Sample:** *N* = 50 (n= 24 intervention; *N* = 26 control) non-cancer patients (57% circulatory, 35% respiratory)	**Intervention:** usual care + SIPS care, including: palliative care assessment needs/concerns; MD review and management; coordination of care for 12 weeks with up to three-visits/contacts. **Control:** usual care (offered SIPS care at 12 weeks)	Intervention had significantly lower symptom distress than control at 6 and 12 weeks (IPOS) Symptom distress reduced with decreased costs for intervention compared to control (i.e., cost-effective) No significant differences between groups in psychosocial concerns (IPOS), ADLs (Barthel), QoL (EQ5D) or burden (ZBI)
IPOS	(90, 91), Ireland	**Design:** Mixed methods, implementation study **Aim:** To test the feasibility and acceptability of using IPOS, with nurse education and training, to improve the identification and management concerns of CHF patients	**Setting:** Nurse-led CHF disease management clinic in two tertiary referral centers **Sample:** *N* = 38 CHF patients (25 retained); 15 caregivers (10 retained)	**Intervention:** Nurse education and training; IPOS completed by patient at clinic visit; nurse assessed/managed needs and symptoms; implementation strategies included reminders, researcher support and staff engagement.	47% Consent rate (372 screened, 81 approached, 38 recruited) 60% IPOS completion rate 6% IPOS items missing ESAS-r, KCCQ, PHQ-8 and ZBI completion feasible via telephone The intervention and study design was feasible and acceptable. Patients and nurses reported supported identification of unmet needs; enabled holistic assessment; empowered patients.
NAT: PD-HF	(61), Netherlands	**Design:** Mixed methods, implementation pilot trial **Aim:** To test the feasibility and acceptability of Dutch NAT: PD-HF in HF outpatients and preliminary effectiveness of patient outcomes and PC referral	**Setting:** Academic hospital **Sample:** *N* = 23 HF outpatients; *N* = 10 carers; *N* = 8 HF nurses	**Intervention:** Nurses were trained in use of tool the NAT: PD-HF Dutch version; tool implemented during routine home care visit; actions taken by nurses in response	Acceptability: medium score of 7/10 (0 = not at all, 10 = very acceptable) Time taken: average 26 minutes 100% patients had PC needs; 11 (48%) actions taken, 4 (17%) were referred to other team/services Barriers/challenges: Difficult to assess PC needs; limited cultural adaptation; lack of prognostic awareness; role confusion; and lack of inter-disciplinary collaboration
RADPAC	(92–94), UK	**Design:** Cluster randomized controlled trial **Aim:** To test impact of GP training in identifying palliative patients and delivering structured, proactive PC.	**Setting: Two** general practices **Sample:** *N* = 159 (*N* = 80 intervention, *N* = 79 control) Cancer, COPD and CHF patients	**Intervention:** GP training in RADPAC, GP coaching session with PC physician in developing care plans, peer group sessions. Complete RADPAC with patients; medical record audit completed. **Control:** standard care; medical record audit completed.	57 GPs completed training in RADPAC No differences between intervention and control Only 50% intervention GPs identified patients (24% of deceased patients) Identified patients – more GP contact and more deaths at home, fewer hospitalisations 1 year later: trained GPs identified more palliative patients than did untrained GPs and delivered multidimensional palliative care
Supportive care decision aid	(95), UK	**Design:** Before and after, implementation study **Aim:** To test the impact of implementing a supportive care decision aid to identify and address unmet palliative and supportive care needs for patients with IPF.	**Setting:** Outpatient (referral ILD center) **Sample:** *N* = 89 (pre) and *N* = 73 (post) (*N* = 64 deceased) IPF patients	**Intervention:** Tool adapted from renal service tool, refined with expert / MDT input and pilot tested; Pre-implementation audit of hospice referrals, mortality data, medical records ILD service; Post-implementation: decision aid trialed for all patients in ILD clinics over 3 months. Same data collected for post cohort as pre cohort.	Completion rate 49%; Tool completion linked to increase in PC referral (17 vs. 3%). Post-implementation: significant increases in documented discussion PC referral (53 vs. 11%), end-of-life discussions (92 vs. 16%).

*AKPS, Australia modified Karnofsky Performance Status; CHF, Chronic Heart Disease; COPD, Chronic Obstructive Pulmonary Disease; GSF, Gold Standards Framework; HF, Heart Failure; ILD, Interstitial Lung Disease; IPF, Idiopathic pulmonary Fibrosis; NAT, PD-HF, Needs Assessment Tool, Progressive Disease – Heart Failure; NECPAL-HF, NECesidades PALiativas; PC, Palliative Care; RADPAC, Radboud indicators for Palliative Care Needs; QoL, Quality of Life; SIPS: short-term integrated palliative and supportive care; SPC, Specialist Palliative Care; UK, United Kingdom*.

### Acceptability, Feasibility and Clinical Utility of Implemented Tools

The feasibility of using tools to identify patients in need of palliative care in a range of settings was explored (Table 2). Tools such as the GSF-PIG (86), SPICT (51) and NECPAL (44, 88) were used to identify the proportion of HF and COPD patients in need of palliative care across general practice, hospital and outpatient settings. A prospective cohort study incorporated the NAT: PD-HF in a battery of assessment tools to test a newly developed definition of need for SPC in patients hospitalized with HF (17). Palliative care needs were identified for 27% of patients, however NAT: PD-HF score alone did not significantly predict PC needs. Utility of the German version of the IPOS was reported in a study of hospitalized HF patients, with 56% patients identified as suitable for palliative care (87).

Five studies examined the implementation of the tool(s) alone or as part of a broader intervention on care processes and services outcomes. A pilot implementation trial of a NAT: PD-HF intervention combined with nurse training did not improve communication about PC needs (61). No improvements in symptom burden, physical functioning, care dependency, or caregiver burden, end of life documentation or health care utilization were recorded, however, the intervention was not adequately powered for efficacy testing. In a mixed-methods, implementation study, use of the IPOS in a HF clinic, supported by nurse education and implementation strategies (reminders, staff engagement and research support), was found to be acceptable and feasible (90). Patients and nurses reported the approach improved recognition of needs, facilitated a more holistic assessment and empowered patients; however, some nurses reported uncertainty when it came to addressing identified needs (91). A small before and after study reported benefits of a shared care pathway and supporting tools for patients with HF, including improved access to palliative care, preferred place of death and access to a holistic HF service from point of care to the end of life (97). A cluster randomized controlled trial involved training GPs in identifying patients in need of palliative care and care planning using the RADPAC (93). Among deceased patients in both study groups, no differences were found for out-of-hours contact, GP contacts, place of death, or hospitalisations (93). However, the sub-group of identified patients had more GP contacts, less hospitalisations and were more likely to die at home. Longer-term outcomes, assessed 12-months later found trained GPs identified more palliative patients (most with a cancer diagnosis) and delivered multidimensional palliative care more often than untrained GPs (94). Another before and after implementation study of a supportive care decision aid with ILD patients found that completion was linked to increase in palliative care referral (17 vs. 3%) (95). Significant increases in documented discussions of palliative care referral (53 vs. 11%) and end-of-life discussions (92 vs. 16%) were reported for the post-implementation cohort. Effectiveness and cost-effectiveness were reported in only one trial of a needs-based palliative and supportive care intervention, with significant reductions in symptom distress (measured by IPOS) of older people living with chronic non-cancer (89).

## Discussion

This systematic review examined the psychometric quality, acceptability and clinical utility of needs assessment tools in identifying and addressing the palliative care needs of people with HF and chronic respiratory diseases. None of the tools included in this review met all psychometric criteria. Evidence for the acceptability and clinical utility of using the tools in these populations in clinical practice is limited.

A two stage process for needs assessment in routine practice has been proposed in the literature (35, 98). The first stage requires a pragmatic method of identifying those who are currently experiencing, or are likely to develop, palliative care needs (35, 98). Brief tools may be most appropriate for this purpose, particularly in busy settings with limited resources. These tools may also be more feasible for these patients, given the expected gradual, abrupt or intermittent functional decline as they progress toward the end of life. However, no tools identified in this review were designed to provide a brief snapshot of the needs of the target population (i.e., <5 min). The IPOS, NAT: PD-HF and NAT: PD-ILD, with an estimated completion time of 10 min, offer opportunity for development in this area. Alternatively, short provider-completed tools, such as the SPICT, NECPAL and RADPAC, may be useful as a first step in identifying those for whom a palliative approach may be beneficial (98–100). Disadvantages of these tools include their generic nature, that they do not quantify the severity or nature of the palliative care needs, and a lack of action prompts to address needs. Instead, these tools focus primarily on disease-related indicators (98, 99). This could result in under-recognition of holistic needs across psychological, social, cultural, and spiritual domains as defined by the WHO (101).

The second stage should involve the use of tools that facilitate a more comprehensive assessment of the nature and severity of needs patients may experience across domains (35, 98, 100). The mode of administration and potential burden remain important considerations for selection. Self-report tools, such as the HFNAQ, CareQol CHF and PNAP, place the individual patient as the expert, potentially promoting a person-centered approach to care. However, some self-report tools may be too burdensome for patients who are facing the end of their life and/or experiencing severe exacerbations. For instance, the estimated completion time for the PNAP is 45 min. Self-report tools are also challenging to implement with patients who are acutely unwell or close to death. Tools that rely on proxy ratings, in contrast, can minimize patient burden, but ratings may not always accurately reflect patients' perceptions of what is most important to them or where they want support. Some tools, such as the IPOS, NAT: PD-HF, I-HARP and NAT: PD-ILD were developed to provide a combination of patient-proxy ratings, either through the completion of different versions of the tool (IPOS) or by completing the tool during consultations with patients and/or family members (NATs and IHARP). While the former enables a comparison of ratings to inform care planning, an advantage of the latter approach is that it enables a real-time discussion of what is most important to the patient, as well as the acceptability of actions that providers may suggest to address identified needs. This, however, has implications for time burden, highlighting the importance of exploring impact on time and resources.

Underpinning the development of needs assessment tools, is the perception that these tools can be feasibily implemented so that patients with identified needs can receive appropriate care, leading to an improvement in outcomes. Our review identified few studies examining the acceptability, feasibility and clinical utility of tools in routine practice. This suggests to date, few data report work in this area for for HF and respiratory disease when compared with measures development and descriptive research. Many were single-center, cross-sectional studies aimed at estimating prevalence. The settings in which these tools were implemented varied considerably, with the majority focused on a heterogeneous population in which people with HF or respiratory diseases comprised a smaller proportion. Data on acceptability from the perspective of the health care team implementing the tool, as well as level of burden and additional support and resources required to successfully implement care plans developed as a consequence, were rarely examined. To date, the RADPAC is the only identification tool which has been tested in a methodologically rigorous controlled trial. Despite being introduced within the context of a multi-component package that included GP education and training, no significant differences were found between the intervention and control group. The finding that a sub-group of identified patients reported more home deaths and fewer hospitalizations, and that trained GPs identified more palliative patients and delivered more palliative care, suggests utility and effectiveness warrants further examination. Organizations such as the European Association for Palliative Care Task Force have recommended the SPICT for use in HF populations (99), however, acknowledge further work is needed to validate this tool.

Most studies involved implementing tools without consideration of actions to be taken to address recognized needs. As part of this, a key challenge for needs-based approach is to determine the most appropriate methods for scoring unmet needs surveys and determining what constitutes a clinically significant change. Further, a lack of education and training for the providers involved was highlighted as an important limitation. For instance, in a Dutch study involving nurses implementing the NAT: PD-HF, nurses reported lacking the knowledge and training to address identified needs (61). In the case of the NAT: PD-HF and NAT: PD-ILD, these actions are largely based on clinical judgement, without clear criteria for referral. Evidence of effectiveness and cost-effectiveness for improving outcomes is also lacking.

### Implications for Research, Practice and Policy

There is emerging evidence that palliative care is an effective approach for people diagnosed with HF and chronic respiratory conditions. Traditional palliative care approaches rely on prognosis and diagnosis as triggers for referral (102). However, the poor utility of available prognostic tools and the ambiguous relationship between prognosis and palliative care needs suggest that prognostication may not be an appropriate trigger (9, 26, 31, 99, 103). Implementing approaches confirmed as efficacious in one patient cohort, such as cancer, and translating them into practice with other non-malignant cohorts is insufficient given their unique burden and complexities (102). A needs-based approach offers a promising alternative, but the rigor of the approach must be established before such processes are accepted and widely implemented. The limited evidence for successful implementation and the psychometric shortcomings of existing tools, demonstrates the importance of psychometrically robust tools to progress the field. Further validation of tools that can reliably and repeatedly assess unmet needs across the broad range of palliative care domains, as well as identify changes in needs over time, is required. The interpretation and utility of these tools with HF and chronic respiratory populations also requires further development of criteria defining clinical significance and clinically important changes in needs.

Identification of needs must also be supported by care processes and actions that are informed by best available evidence, align with needs and do not cause undue harm. Structured care processes (e.g., care bundles) potentially have numerous benefits for delivering good clinical care, while also facilitating measurement and feedback processes (104, 105). Studies quantifying the nature, severity and trajectory of unmet needs for HF and chronic respiratory conditions can inform the selection of care processes with which to intervene. Generalist and specialist providers should receive targeted education and training to ensure they are equipped with the skills to: recognize palliative care needs; appropriately communicate this with patients; and provide appropriate care (102). Promoting earlier identification of palliative care needs and appropriate care planning, tailored to medical conditions, has the potential to achieve hospital avoidance, death in place of choice, better symptom control and less family distress. Improvements in planning and clinical care can also potentially reduce the distress experienced by health professionals in this field.

### Study Limitations

A strength of this review include the systematic literature search that encompassed a wide range of broad search terms and multiple databases. However, gray literature, dissertations or policy documents were not included, as while this literature contributes important information, it is not peer-reviewed. Publications were also restricted to English language, which may have resulted in some studies being missed.

## Conclusion

The impetus to develop and implement tools for palliative care is reflected in the increasing number of needs assessment tools being developed and tested with HF and chronic respiratory disease populations. However, further evidence of psychometric quality is needed, particularly test-retest reliability, predictive validity, responsiveness, and clinical utility of these tools. Further, relying on “needs” as the recommended criterion must be supported by a systematic approach that incorporates structured care processes; improved community awareness of the potential benefits offered by palliative care; and education and training for providers across care settings. Rigorous evaluation to determine the impact of adopting a systematic needs-based approach for HF and chronic respiratory disease on the physical and psychosocial outcomes of patients and carers, as well as the economic costs and benefits to the healthcare system, is required.

## Data Availability Statement

The original contributions presented in the study are included in the article/Supplementary Material, further inquiries can be directed to the corresponding author.

## Author Contributions

AW: conceptualization. AW, KF, and BH: screening of articles and data extraction (methodology). AW, BH, KF, and KC: analysis, interpretation, and writing (original draft preparation). All authors contributed to the final version of the article and approved the submitted version.

## Funding

BH is supported by a Colin Dodds Australian Rotary Health Postdoctoral Fellowship (G1801108). This research was supported by the National Health and Medical Research Council via a Dementia Research Team grant (1095078) and also infrastructure funding from the University of Newcastle and Hunter Medical Research Institute.

## Conflict of Interest

The authors declare that the research was conducted in the absence of any commercial or financial relationships that could be construed as a potential conflict of interest.

## Publisher's Note

All claims expressed in this article are solely those of the authors and do not necessarily represent those of their affiliated organizations, or those of the publisher, the editors and the reviewers. Any product that may be evaluated in this article, or claim that may be made by its manufacturer, is not guaranteed or endorsed by the publisher.
