# Supplementary material for: A Systematic Review of the Development and Implementation of Needs-Based Palliative Care Tools in Heart Failure and Chronic Respiratory Disease
Source: Front Cardiovasc Med. 2022 Apr 13;9:878428. doi: 10.3389/fcvm.2022.878428 (PMC9043454; doi:10.3389/fcvm.2022.878428)
Supplement: Supplementary file 1 [file Table_1.DOC]

Database: Ovid MEDLINE(R) and Epub Ahead of Print, In-Process, In-Data-Review & Other Non-Indexed Citations and Daily <1946 to November 05, 2021>

Search Strategy:

--------------------------------------------------------------------------------

1 palliative care/ or terminal care/ or hospice care/ (84374)

2 terminally ill/ (6706)

3 (palliativ* or terminal* ill* or terminal care or supportive care).ti,ab,kw,kf. (95229)

4 ("end of life" or "end-of-life").ti,ab. (25417)

5 1 or 2 or 3 or 4 (147607)

6 needs assessment/ or assessment.ti,ab,kw,kf. (1121912)

7 "health services needs and demand"/ (54616)

8 patient outcome assessment/ or symptom assessment/ or patient reported outcome measures/ (21680)

9 (need* adj2 (assess* or unmet or perceived or support* or patient*)).ti,ab,kw,kf. (141449)

10 6 or 7 or 8 or 9 (1296197)

11 exp heart failure/ (132858)

12 cardiovascular diseases/ or cardiomyopathies/ or ventricular dysfunction, left/ (218169)

13 (heart failure or left ventricular or cardiovascular or cardiomyopath*).ti,ab,kw,kf. (815946)

14 ((heart* or cardiac* or myocard*) adj2 (fail* or insuff* or decompensation)).ti,ab,kw,kf. (213199)

15 11 or 12 or 13 or 14 (913037)

16 exp pulmonary disease, chronic obstructive/ (60991)

17 cystic fibrosis/ (37337)

18 (chronic adj2 (pulmonary or lung*)).ti,ab,kw,kf. (81185)

19 COPD.ti,ab,kw,kf. (51982)

20 ((end stage or advanced) adj3 (lung or pulmonary or cystic fibrosis)).ti,ab,kw,kf. (8950)

21 exp lung diseases, interstitial/ (58701)

22 exp lung diseases, obstructive/ (223079)

23 exp pulmonary fibrosis/ (25496)

24 emphysem*.ti,ab,kw,kf. (29227)

25 (chronic* adj3 bronchiti*).ti,ab,kw,kf. (11491)

26 (obstruct* adj3 (pulmonary or lung* or airway* or airflow* or bronch* or respirat*)).ti,ab,kw,kf. (96056)

27 COAD.ti,ab,kw,kf. (613)

28 (interstitial* adj3 (lung* adj3 disease*)).ti,ab,kw,kf. (13213)

29 (interstitial* adj3 (fibros* or pneumonitis or pneumonia or pneumopathy)).ti,ab,kw,kf. (23131)

30 alveolitis.ti,ab,kw,kf. (4242)

31 exp bronchiolitis obliterans/ (3963)

32 (bronchiolitis adj3 obliterans).ti,ab,kw,kf. (3802)

33 exp Pneumoconiosis/ or (pneumoconios?s or pneumonoconiosis).ti,ab,kw,kf. (21398)

34 16 or 17 or 18 or 19 or 20 or 21 or 22 or 23 or 24 or 25 or 26 or 27 or 28 or 29 or 30 or 31 or 32 or 33 (439745)

35 15 or 34 (1331904)

36 5 and 10 and 35 (1071)

37 limit 36 to (english language and yr="2012 -Current") (694)

38 limit 37 to (case reports or comment or editorial or letter or news) (30)

39 37 not 38 (664)

Database: Embase <1947 to present>

Search Strategy:

--------------------------------------------------------------------------------

1 terminal care/ or hospice care/ (48307)

2 palliative therapy/ or cancer palliative therapy/ (126682)

3 terminally ill patient/ or hospice patient/ (9131)

4 (palliativ* or terminal* ill or terminal care or supportive care or "end of life").ti,ab,kw,kf. (178106)

5 1 or 2 or 3 or 4 (245453)

6 needs assessment/ or assessment.ti,ab,kw,kf. (1617190)

7 health care need/ (32746)

8 symptom assessment/ (9635)

9 patient reported outcome/ (34677)

10 (need* adj2 (assess* or unmet or perceived or support* or patient*)).ti,ab,kw,kf. (225138)

11 6 or 7 or 8 or 9 or 10 (1855213)

12 exp heart failure/ (586382)

13 cardiovascular disease/ (302309)

14 exp cardiomyopathy/ (157951)

15 exp heart ventricle function/ (73212)

16 (heart failure or left ventricular or cardiovascular or cardiomyopath*).ti,ab,kw,kf. (1251276)

17 ((heart* or cardiac* or myocard*) adj2 (fail* or insuff* or decompensation)).ti,ab,kw,kf. (361187)

18 12 or 13 or 14 or 15 or 16 or 17 (1619983)

19 chronic obstructive lung disease/ (151167)

20 cystic fibrosis/ (80313)

21 (chronic adj2 (pulmonary or lung)).ti,ab,kw,kf. (123371)

22 COPD.ti,ab,kw,kf. (98795)

23 ((end stage or advanced) adj3 (lung or pulmonary or cystic fibrosis)).ti,ab,kw,kf. (15913)

24 exp interstitial lung disease/ (96518)

25 obstructive lung disease/ (140)

26 exp lung fibrosis/ (91995)

27 emphysem*.ti,ab,kw,kf. (46369)

28 (chronic* adj3 bronchiti*).ti,ab,kw,kf. (18503)

29 (obstruct* adj3 (pulmonary or lung* or airway* or airflow* or bronch* or respirat*)).ti,ab,kw,kf. (146551)

30 COAD.ti,ab,kw,kf. (760)

31 (interstitial* adj3 (lung* adj3 disease*)).ti,ab,kw,kf. (25073)

32 (interstitial$ adj3 (fibros* or pneumonitis or pneumonia or pneumopathy)).ti,ab,kw,kf. (37055)

33 alveolitis.ti,ab,kw,kf. (6092)

34 bronchiolitis obliterans/ (6207)

35 (bronchiolitis adj3 obliterans).ti,ab,kw,kf. (6384)

36 (pneumoconios?s or pneumonoconiosis).ti,ab,kw,kf. (7377)

37 19 or 20 or 21 or 22 or 23 or 24 or 25 or 26 or 27 or 28 or 29 or 30 or 31 or 32 or 33 or 34 or 35 or 36 (535755)

38 18 or 37 (2098320)

39 5 and 11 and 38 (2714)

40 limit 39 to (english language and yr="2012 -Current") (2020)

41 limit 40 to (books or chapter or conference abstract or conference paper or "conference review" or editorial or letter or note) (999)

42 40 not 41 (1021)

Database: APA PsycInfo <1806 to November Week 1 2021>

Search Strategy:

--------------------------------------------------------------------------------

1 Palliative care/ or Terminally ill/ or Terminal cancer/ (17089)

2 (palliativ* or terminal* ill or terminal care or supportive care).ti,ab,id. (16955)

3 "end of life".ti,ab,id. (10537)

4 1 or 2 or 3 (24902)

5 Needs assessment/ or Health service needs/ (10712)

6 assessment.ti,ab,id. (335472)

7 (need* adj2 (assess* or unmet or perceived or support* or patient*)).ti,ab,id. (43840)

8 5 or 6 or 7 (373845)

9 exp heart disorders/ (15152)

10 (heart failure or left ventricular or cardiovascular or cardiomyopath*).ti,ab,id. (35939)

11 ((heart* or cardiac* or myocard*) adj2 (fail* or insuff* or decompensation)).ti,ab,id. (4426)

12 exp lung disorders/ (4782)

13 (chronic adj2 (pulmonary or lung)).ti,ab,id. (2961)

14 COPD.ti,ab,id. (1754)

15 ((end stage or advanced) adj3 (lung or pulmonary or cystic fibrosis)).ti,ab,id. (284)

16 emphysem*.ti,ab,id. (268)

17 (chronic* adj3 bronchiti*).ti,ab,id. (154)

18 (obstruct* adj3 (pulmonary or lung* or airway* or airflow* or bronch* or respirat*)).ti,ab,id. (3072)

19 COAD.ti,ab,id. (21)

20 (interstitial* adj3 (lung* adj3 disease*)).ti,ab,id. (55)

21 (interstitial* adj3 (fibros* or pneumonitis or pneumonia or pneumopathy)).ti,ab,id. (52)

22 alveolitis.ti,ab,id. (6)

23 (bronchiolitis adj3 obliterans).ti,ab,id. (9)

24 (pneumoconios?s or pneumonoconiosis).ti,ab,id. (22)

25 9 or 10 or 11 or 12 or 13 or 14 or 15 or 16 or 17 or 18 or 19 or 20 or 21 or 22 or 23 or 24 (52336)

26 4 and 8 and 25 (206)

27 limit 26 to (english language and yr="2012 -Current") (120)

28 limit 27 to (chapter or "column/opinion" or "comment/reply" or editorial or letter) (8)

29 27 not 28 (112)

Search Name: Amy COPD pall care

Date Run: 08/11/2021 07:21:44

Comment:

ID Search Hits

#1 MeSH descriptor: [Palliative Care] this term only 1709

#2 MeSH descriptor: [Terminal Care] this term only 366

#3 MeSH descriptor: [Hospice Care] this term only 110

#4 MeSH descriptor: [Terminally Ill] this term only 92

#5 ("end of life" or end-of-life or palliativ* or "terminal illness" or "terminally ill" or "terminal care" or "supportive care"):ti,ab,kw 12293

#6 #1 or #2 or #3 or #4 or #5 12326

#7 MeSH descriptor: [Needs Assessment] this term only 366

#8 assessment:ti,ab,kw 253880

#9 MeSH descriptor: [Health Services Needs and Demand] this term only 350

#10 MeSH descriptor: [Symptom Assessment] this term only 289

#11 MeSH descriptor: [Patient Outcome Assessment] this term only 315

#12 MeSH descriptor: [Patient Reported Outcome Measures] this term only 838

#13 need* near/2 (assess* or unmet or perceived or support* or patient*):ti,ab,kw 20052

#14 #7 or #8 or #9 or #10 or #11 or #12 or #13 268760

#15 MeSH descriptor: [Heart Failure] explode all trees 10029

#16 MeSH descriptor: [Cardiovascular Diseases] this term only 8694

#17 MeSH descriptor: [Cardiomyopathies] this term only 681

#18 MeSH descriptor: [Ventricular Dysfunction, Left] this term only 1997

#19 ("heart failure" or "left ventricular" or cardiovascular or cardiomyopath*):ti,ab,kw 110918

#20 #15 or #16 or #17 or #18 or #19 111073

#21 MeSH descriptor: [Pulmonary Disease, Chronic Obstructive] explode all trees 6098

#22 MeSH descriptor: [Cystic Fibrosis] this term only 1861

#23 (chronic Near/2 (pulmonary or lung*)):ti,ab,kw 18829

#24 COPD:ti,ab,kw 17319

#25 (("end stage" or advanced) Near/3 (lung or pulmonary or cystic fibrosis)):ti,ab,kw 1922

#26 MeSH descriptor: [Lung Diseases, Interstitial] explode all trees 816

#27 MeSH descriptor: [Lung Diseases, Obstructive] explode all trees 20326

#28 MeSH descriptor: [Pulmonary Fibrosis] explode all trees 604

#29 emphysem*:ti,ab,kw 1605

#30 (chronic* Near/3 bronchiti*):ti,ab,kw 2055

#31 (obstruct* Near/3 (pulmonary or lung* or airway* or airflow* or bronch* or respirat*)):ti,ab,kw 21218

#32 COAD:ti,ab,kw 87

#33 (interstitial* Near/3 (lung* adj3 disease*)):ti,ab,kw 1472

#34 (interstitial* Near/3 (fibros* or pneumonitis or pneumonia or pneumopathy)):ti,ab,kw 896

#35 alveolitis:ti,ab,kw 840

#36 MeSH descriptor: [Bronchiolitis Obliterans] explode all trees 68

#37 (bronchiolitis Near/3 obliterans):ti,ab,kw 200

#38 MeSH descriptor: [Pneumoconiosis] explode all trees 100

#39 (pneumoconios?s or pneumonoconiosis):ti,ab,kw 76

#40 #21 or #22 or #23 or #24 or #25 or #26 or #27 or #28 or #29 or #30 or #31 or #32 or #33 or #34 or #35 or #36 or #37 or #38 or #39 48337

#41 #20 or #40 156482

#42 #6 and #14 and #41 360 10 Reviews 350 Trials
